# ﻿First record of the genus *Passiena* (Araneae, Lycosidae) from China, with the first description of the male of *P.spinicrus* Thorell, 1890 from Malaysia

**DOI:** 10.3897/zookeys.1182.109532

**Published:** 2023-10-11

**Authors:** Bing Tan, Muhammad Irfan, Zhi-Sheng Zhang, Lu-Yu Wang

**Affiliations:** 1 Key Laboratory of Eco-environments in Three Gorges Reservoir Region (Ministry of Education), School of Life Science, Southwest University, Chongqing 400715, China Southwest University Chongqing China

**Keywords:** New record, new species, morphology, taxonomy, wolf spider

## Abstract

The genus *Passiena* is recorded for the first time from China with *Passienaduani***sp. nov.** (♂♀) from Guangxi described here. In addition, the male of *P.spinicrus* Thorell, 1890 is described for the first time based on a specimen from Malaysia and colour photographs of freshly collected material are also presented. Detailed morphological descriptions, photographs, genital illustrations, and a distribution map for the two species are provided.

## ﻿Introduction

Lycosidae is the sixth largest spider family with 2462 species in 132 genera distributed worldwide, including 310 species in 26 genera reported from China (World Spider Catalog 2023). In recent years, we have described several new genera of wolf spider from China, such as *Serratacosa* Wang, Peng & Zhang, 2021, *Sinacosa* Wang, Lu & Zhang, 2023 and *Sinartoria* Wang, Framenau & Zhang, 2021. Still, most of the lycosid diversity in China has not been fully documented.

The genus *Passiena* Thorell, 1890 contains five species from Cameroon, Indonesia, Laos, Malaysia, South Africa and Thailand (World Spider Catalog 2023). It is diagnosed by the male pedipalp with a unique group of soft spicules on the distal part of the palea (Lehtinen 2005). In this paper, *Passiena* is recorded for the first time in China, and the male of *P.spinicrus* Thorell, 1890, newly found in Malaysia, is described here. *Passienaduani* sp. nov. is predominantly found in the terrestrial habitat beneath forest canopies in Guangxi Province.

## ﻿Materials and methods

All specimens were preserved in 75% ethanol and examined, illustrated, photographed and measured using a Leica M205A stereomicroscope equipped with a drawing tube, a Leica DFC450 camera, and Leica Application Suite software (Ver. 4.6). Male pedipalps and epigynes were examined and illustrated after dissection. Epigynes were cleared in pancreatin (Álvarez-Padilla and Hormiga 2007). Leg measurements are shown as: total length (femur, patella+tibia, metatarsus, tarsus). All measurements are in millimetres. Map was created using the online mapping software SimpleMappr (Shorthouse 2010) (Fig. 5). Specimens examined here are deposited in the spider collection at the School of Life Sciences, Southwest University, Chongqing, China (**SWUC**).

Abbreviations used in the text and figures: **ALE**–anterior lateral eye; **AME**–anterior median eye; **PLE**–posterior lateral eye; **PME**–posterior median eye; **A**–atrium; **Ap**–anterior apophysis of palea; **CO**–copulatory opening; **C**–conductor; **E**–embolus; **FD**–fertilization duct; **HS**–head of spermatheca; **H**–hood; **MA**–median apophysis; **Pt**–tip of posterior apophysis; **St**–subtegulum; **TA**–terminal apophysis; **T**–tegulum; **Se**–septum; **SS**–stalk of spermatheca.

## ﻿Taxonomy

**Family Lycosidae Sundevall, 1833**

**Genus *Passiena* Thorell, 1890** (帕狼蛛属)

### ﻿ Passiena duani

Taxon classificationAnimaliaAraneaeLycosidae

sp. nov. (段氏帕狼蛛)

44FD99A2-F8E6-50F9-A741-F6A7BB7A6D98

https://zoobank.org/6CE69B00-418B-4697-B35E-C2347209460E

**Figure 1.**
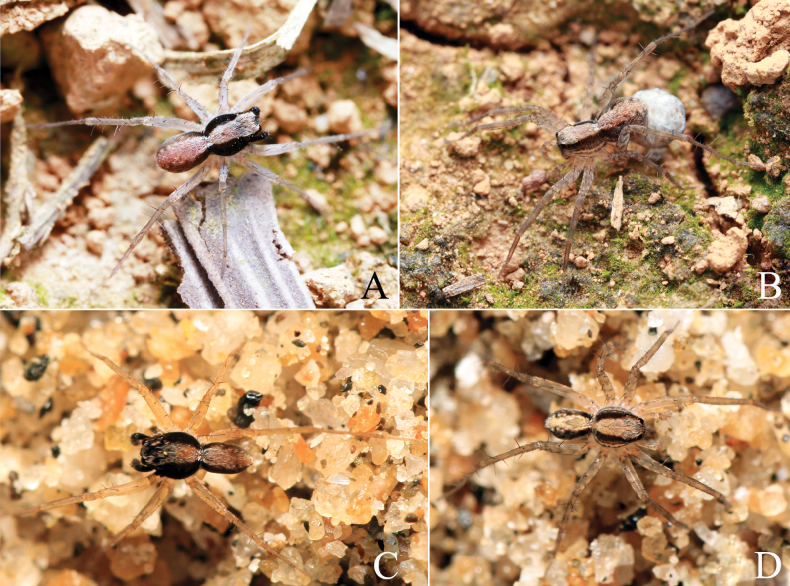
Live photo of *Passienaduani* sp. nov. (**A, B**) and *P.spinicrus* Thorell, 1890 (**C, D**) **A, C** male **B, D** female. Live photos taken by Qian-Le Lu (**A, B**) and Lu-Yu Wang (**C, D**).

**Figure 2.**
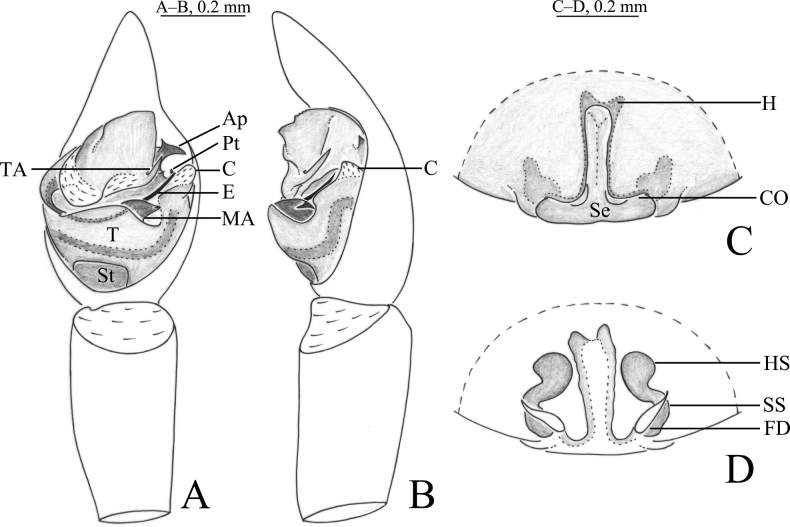
*Passienaduani* sp. nov., male holotype (**A, B**) and female paratype (**C, D**) **A** pedipalp, ventral view **B** same, retrolateral view **C** epigyne, ventral view **D** same, dorsal view.

**Figure 3.**
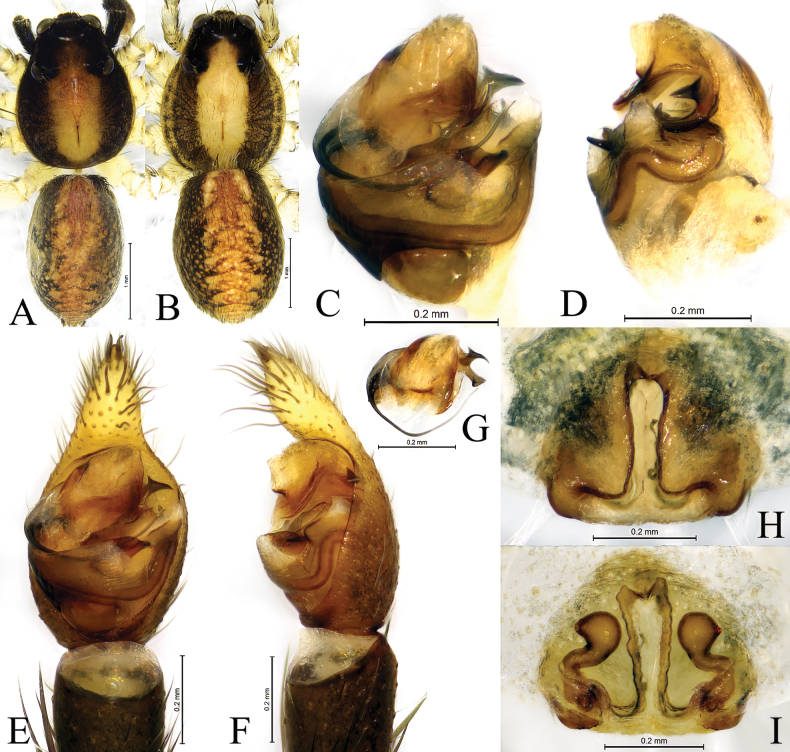
*Passienaduani* sp. nov., male holotype (**A, C–G**) and female paratype (**B, H, I**) **A** male habitus, dorsal view **B** female habitus, dorsal view **C, E** pedipalp, ventral view **D, F** same, retrolateral view **G** embolus and terminal apophysis, ventral view **H** epigyne, ventral view **I** same, dorsal view.

Figs 1A, B , 2A–D , 3A–I , 5

#### Type material.

***Holotype*** (male): China, Guangxi Zhuang Autonomous Region, Chongzuo City, Ningming County, Chengzhong Town, Panlong, 22.2347°N, 107.0538°E, elev. 138 m, 25 April 2023, L.Y. Wang and Q.L. Lu leg. (SWUC-T-LY-13-01); ***Paratypes*** (3 males and 4 females): 2 males and 3 females, same data as holotype; 1 male and 1 female (SWUC-T-LY-13-07~08), Ningming County, Tuolong Township, Nongna Village, 22.2325°N, 107.0558°E, elev. 152 m, 19 June 2017, L.Y. Wang and R.B. Wu leg. (SWUC-T-LY-13-02~06).

#### Etymology.

The specific name comes from the family name of Dr Meichun Duan, who gave much support to our research on spiders; noun in apposition.

#### Diagnosis.

**Figure 4.**
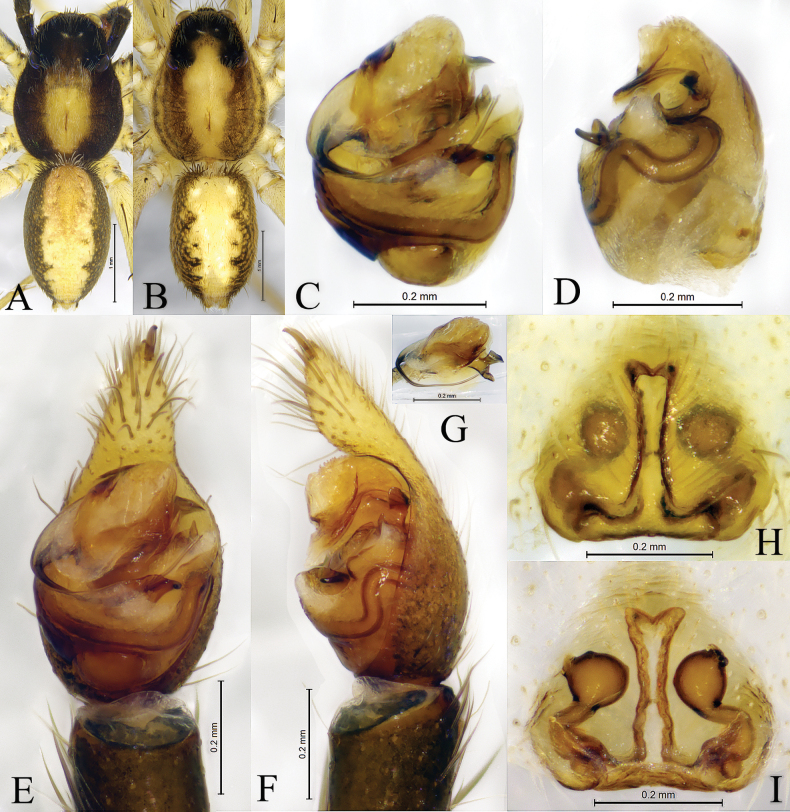
*Passienaspinicrus* Thorell, 1890, male (**A, C–G**) and female (**B, H, I**). **A** male habitus, dorsal view **B** female habitus, dorsal view **C** bulb, ventral view **D** same, retrolateral view **E** pedipalp, ventral view **F** same, retrolateral view **G** embolic division, ventral view **H** epigyne, ventral view **I** same, dorsal view.

The new species resembles *P.bayi* Omelko & Marusik, 2020, *P.torbjoerni* Lehtinen, 2005 (Figs 2A–D, 3C–I; Omelko and Marusik 2020, figs 19–29) and *P.spinicrus* Thorell, 1890 (4C–I) in having similar median apophysis and terminal apophysis of the male pedipalp and variable sclerotization of the lateral plates at the base of the epigyne (Figs 2A–D, 3C–I, 4C–I; Omelko and Marusik 2020, figs 30–35). However, it can be distinguished by the combination of the following characters: 1) apical edge of anterior apophysis of palea as long as the stalk of posterior apophysis of palea (Fig. 3G) vs. about half the length of the stalk of posterior apophysis of palea in *P.bayi*, *P.torbjoerni* and *P.spinicrus* (Omelko and Marusik 2020, figs 25, 26; Fig. 4G); 2) cymbium apex with two claws (Fig. 3G) vs. with one claw in *P.bayi*, *P.torbjoerni* and *P.spinicrus* (Omelko and Marusik 2020, figs 27, 28; Fig. 4G); and 3) epigynal septum stem length/septum base width ratio 1.2 in *P.duani* sp. nov. and *P.torbjoerni* (Figs 2C, D, 3H, I; Omelko and Marusik 2020, figs 33, 34) vs. septum stem length/septum base width ratio 1.5 in *P.bayi* (Omelko and Marusik 2020, figs 30, 31) and *P.spinicrus* (Fig. 4H, I).

#### Description.

**Male** (holotype, Fig. 1A, 3A). Total length 4.08. Prosoma 2.05 long, 1.59 wide; opisthosoma 1.98 long, 1.30 wide. Carapace greyish brown. Eye sizes and interdistances: AME 0.09, ALE 0.07, PME 0.30, PLE 0.25; AME–AME 0.09, AME–ALE 0.04, PME–PME 0.33, PME–PLE 0.32. Clypeus height 0.18. Chelicerae dark brown, with three promarginal and three retromarginal teeth. Endites and labium dark brown, longer than wide. Sternum yellow brown, with sparse brown setae. Legs yellow brown. Tibia I with six pairs of ventral spines and metatarsus I with four pairs of ventral spines; tibia II with five pairs of ventral spines, metatarsus II with three pairs of ventral spines. Leg measurements: I 6.14 (1.63, 2.21, 1.48, 0.82); II 5.47 (1.54, 1.79, 1.36, 0.78); III 5.23 (1.46, 1.57, 1.43, 0.77); IV 8.02 (2.07, 2.38, 2.49, 1.08). Leg formula: 4123. Opisthosoma oval. Dorsum greyish brown, with black markings. Venter yellow brown.

***Pedipalp*** (Figs 2A, B, 3C–G): Cymbium proximal part brown, distal part yellowish with two large claws on the tip. Subtegulum distinct in ventral view, located baso-prolaterally. Conductor somewhat membranous, somewhat tongue-shaped in ventral view and triangular in retrolateral view. Terminal apophysis terminates at approx. 1 o’clock position in ventral view. Embolus originating on the dorsal side of the bulb, long, prolaterally accompanied with a membrane, terminating at approx. 2 o’clock position; palea with two apophyses, anterior apophysis with smooth apical edge sharply pointed, and posterior one claw-like.

**Female** (one paratype, SWUC-T-LY-13-02, Fig. 1B, 3B). Total length 4.57. Prosoma 2.36 long, 1.83 wide; opisthosoma 2.28 long, 1.65 wide. Eye sizes and interdistances: AME 0.12, ALE 0.09, PME 0.35, PLE 0.27; AME–AME 0.10, AME–ALE 0.07, PME–PME 0.33, PME–PLE 0.38. Clypeus height 0.23. Leg measurements: I 6.87 (1.92, 2.43, 1.65, 0.87); II 6.12 (1.58, 2.18, 1.53, 0.83); III 5.96 (1.68, 1.77, 1.70, 0.81); IV 9.28 (2.28, 2.75, 2.98, 1.27). Leg formula: 4123. Tibia I with six pairs of ventral spines and metatarsus I with four pairs of ventral spines; tibia II with six pairs of ventral spines, metatarsus II with four pairs of ventral spines. Except genitalia, all other morphological characteristics same as in male.

***Epigyne*** (Figs 2C–D, 3H–I). Anterior pocket with 2 hoods, septum reversed T-shaped with distinct stem becoming very thick in its anterior part and narrow posteriorly; stem 1.2 times longer than base width. Copulatory openings located posteriorly at the base of atrium transverse edges. Spermathecal heads sub-oval with the antero-lateral part angled, heads 2 times longer than septum base. Spermathecal stalks thick, short, slightly curved. Fertilization ducts teardrop-shaped.

#### Distribution.

**Figure 5.**
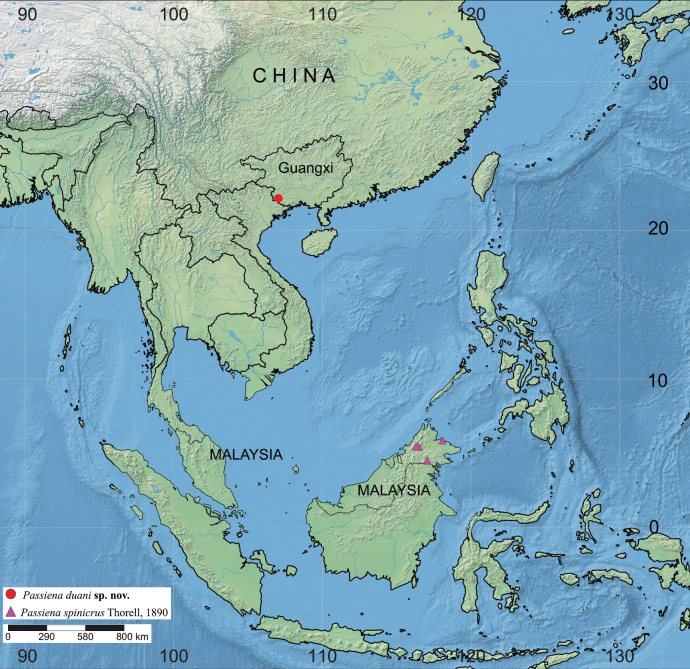
Map showing distribution records of *Passienaduani* sp. nov. and *P.spinicrus* Thorell, 1890.

Currently known only from the type locality, Ningming County, Guangxi, China (Fig. 5).

### ﻿ Passiena spinicrus

Taxon classificationAnimaliaAraneaeLycosidae

Thorell, 1890

5555E6EC-B4A3-51D7-B1B5-8BF01259E443

Figs 1C, D , 4 , 5

Passiena spinicrus Thorell, 1890: 140 (♀); Lehtinen 2005: 402, figs 5–10 (♀); Omelko and Marusik 2020: 480, figs 6, 15, 18, 35 (♀).

#### Material examined.

Malaysia: 6 males and 4 females, Borneo, Sabah, Trus Madi Mountain, 5.4669°N, 116.4488°E, elev. 760 m, 12 October 2015, G.Q. Huang and L.Y. Wang leg. (MLXY-15-15); 2 males and 1 female, Borneo, Sabah, Keningau, apin-apin, 5.4669°N, 116.2752°E, elev. 346 m, 17 October 2015, G.Q. Huang and L.Y. Wang leg. (MLXY-15-25); 4 males and 5 females, Borneo, Kalabakan, Maliau Basin, 4.54°N, 117.0272°E, elev. 321 m, 18 October 2015, G.Q. Huang and L.Y. Wang leg. (MLXY-15-29); 1 male and 1 female, Borneo, Sabah, Sandakan, 5.8788°N, 118.0536°E, elev. 41 m, 19 October 2015, G.Q. Huang and L.Y. Wang leg. (MLXY-15-35).

#### Description.

**Male** (Figs 1C, 4A) total length 3.91. Prosoma 2.16 long, 1.61 wide; opisthosoma 1.74 long, 1.02 wide. Carapace gray brown. Eye sizes and interdistances: AME 0.12, ALE 0.08, PME 0.34, PLE 0.28; AME–AME 0.10, AME–ALE 0.06, PME–PME 0.34, PME–PLE 0.37. Clypeus height 0.24. Chelicerae black brown, with three promarginal and three retromarginal teeth. Endites and labium black brown, longer than wide. Sternum yellow brown, with sparse brown hairs. Legs yellow brown. Tibia I with six pairs of ventral spines and metatarsus I with four pairs of ventral spines; tibia II with six pairs of ventral spines, metatarsus II with four pairs of ventral spines. Leg measurements: I 6.63 (1.65, 2.39, 1.69, 0.90); II 5.98 (1.66, 1.99, 1.54, 0.79); III 5.75 (1.55, 1.82, 1.60, 0.78); IV 8.87 (2.22, 2.59, 2.87, 1.19). Leg formula: 4123. Opisthosoma oval. Dorsum greyish brown, with black markings. Venter yellow brown.

***Pedipalp*** (Fig. 4C–G): Cymbium proximal part brown, distal part yellowish with two large claws on the tip. Subtegulum distinct in ventral view, located baso-prolaterally. Conductor somewhat membranous and tongue-shaped in ventral view and triangular in retrolateral view. Terminal apophysis terminates at approx. 1 o’clock position in ventral view. Embolus originating on the dorsal side of the bulb, long, prolaterally accompanied with a membrane, terminating at approx. 2 o’clock position; palea with two apophyses, anterior apophysis ax-shaped, and the posterior apex strongly curved.

**Female** (Figs 1D, 4B) total length 4.21. Prosoma 2.20 long, 1.77 wide; opisthosoma 1.89 long, 1.23 wide. Eye sizes and interdistances: AME 0.12, ALE 0.09, PME 0.33, PLE 0.29; AME–AME 0.08, AME–ALE 0.05, PME–PME 0.32, PME–PLE 0.37. Clypeus height 0.21. Legs yellow brown. Tibia I with six pairs of ventral spines and metatarsus I with four pairs of ventral spines; tibia II with six pairs of ventral spines, metatarsus II with three pairs of ventral spines. Leg measurements: I 6.96 (1.92, 2.56, 1.66, 0.82); II 6.36 (1.77, 2.19, 1.57, 0.83); III 6.02 (1.64, 1.89, 1.68, 0.81); IV 9.04 (2.38, 2.56, 2.87, 1.23). Leg formula: 4123.

***Epigyne*** (Fig. 4H, I). Anterior pocket with 2 hoods, septum reversed T-shaped with distinct stem becoming very thick in its anterior part and narrow at the center. Copulatory openings located posteriorly at the base of atrium transverse edges. Spermathecal heads globular with the antero-lateral part angled, heads 2 times longer than septum base. Spermathecal stalks thick, short, slightly curved. Fertilization ducts extending postero-laterally.

#### Distribution.

Malaysia, Indonesia (Borneo) (Fig. 5).

## ﻿Discussion

In all known species of the genus *Passiena*, it is observed that the reproductive organs of both male and female individuals exhibit a remarkable resemblance, hence posing a considerable challenge in terms of distinguishing between congeners. The majority of species can be distinguished solely based on the morphology of the palea apophyses in the male pedipalps and the spermathecae shape in epigynes as can be seen in the study carried out by Omelko and Marusik (2020). Logunov and Ponomarev (2020) and Lehtinen (2005) used morphological traits to place this genus into the subfamily Lycosinae. However, no molecular analysis of *Passiena* was included in the largest phylogenetic analysis of Lycosidae by Piacentini and Ramírez (2019). It is highly recommended that future studies undertake a revision of *Passiena*, taking into consideration both molecular and morphological data.

## Supplementary Material

XML Treatment for Passiena duani

XML Treatment for Passiena spinicrus

## ﻿Additional information

### Author ORCIDs

Bing Tan https://orcid.org/0009-0003-0517-1116

Muhammad Irfan https://orcid.org/0000-0003-0445-9612

Zhi-Sheng Zhang https://orcid.org/0000-0002-9304-1789

Lu-Yu Wang https://orcid.org/0000-0002-5250-3473

### Data availability

All of the data that support the findings of this study are available in the main text.
